# Wrapped Left Anterior Descending Artery Presenting As Inferior Myocardial Infarction: Case Report and Review of the Literature

**DOI:** 10.7759/cureus.13358

**Published:** 2021-02-15

**Authors:** Ali R Ghani, Mohsin S Mughal, Sundeep Kumar, Preetham Muskula, Elsayed Abo-Salem

**Affiliations:** 1 Department of Cardiology, Saint Louis University Hospital, St. Louis, USA; 2 Department of Internal Medicine, Monmouth Medical Center, Long Branch, USA

**Keywords:** inferior wall myocardial infarction, st-elevation myocardial infarction (stemi)

## Abstract

Acute occlusion of the left anterior descending (LAD) coronary artery generally results in ST-segment elevation in the anterior leads of the electrocardiogram and reciprocal ST-segment depression in the inferior leads. We present a case of LAD occlusion presenting as inferior wall ST-segment elevation myocardial infarction.

## Introduction

Electrocardiogram (ECG) is an important and accessible tool in the detection of acute myocardial infarction (MI). ECG changes in various leads point towards the culprit coronary artery [1,2]. Inferior ST-segment elevation MI (STEMI) is usually caused by occlusion of the right coronary artery (RCA) or less commonly, left circumflex artery (LCX). Similarly, precordial lead changes, especially V1, V2, and V3, usually point towards LAD occlusion. LCX occlusion is usually electrically silent on ECG. Concomitant anterior and inferior MI has been reported to be due to occlusion of a “wrapped LAD” [3]. We present a case of LAD occlusion presenting as inferior wall STEMI on ECG.

## Case presentation

A 51-year-old woman with a past medical history of hypertension and diabetes presented to the emergency department with sudden onset chest pain radiating to both shoulders; her pain was 10/10 on the visual analog scale. The pain woke her up from sleep and was associated with diaphoresis and nausea. She took 325 mg of aspirin at home before the arrival of emergency medical services. The pain was still present on admission and unchanged from the time of onset.

**Figure 1:**
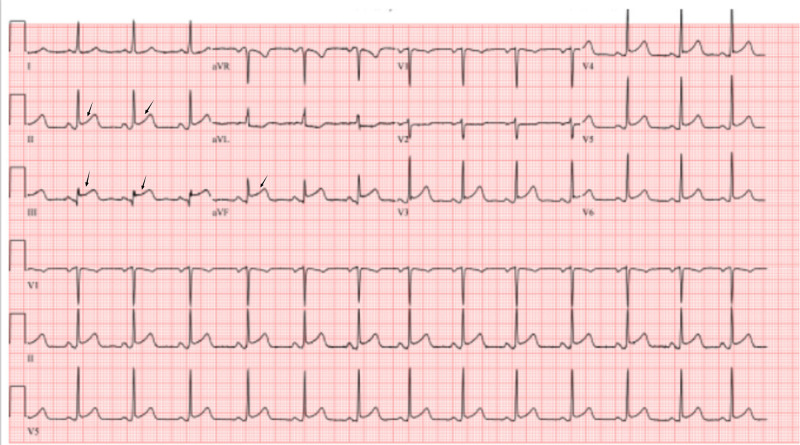
ECG showing STEMI in the inferior leads. Abbreviations: ECG, electrocardiogram; STEMI, ST-segment elevation myocardial infarction.

Initial ECG showed ST-segment elevation in the inferior leads (Figure 1), and the patient was taken to the cardiac catheterization laboratory for revascularization. Initial vital signs included a blood pressure of 184/89 mmHg, a pulse of 112 beats/minute, and a respiratory rate of 20 breaths per minute.

**Figure 2:**
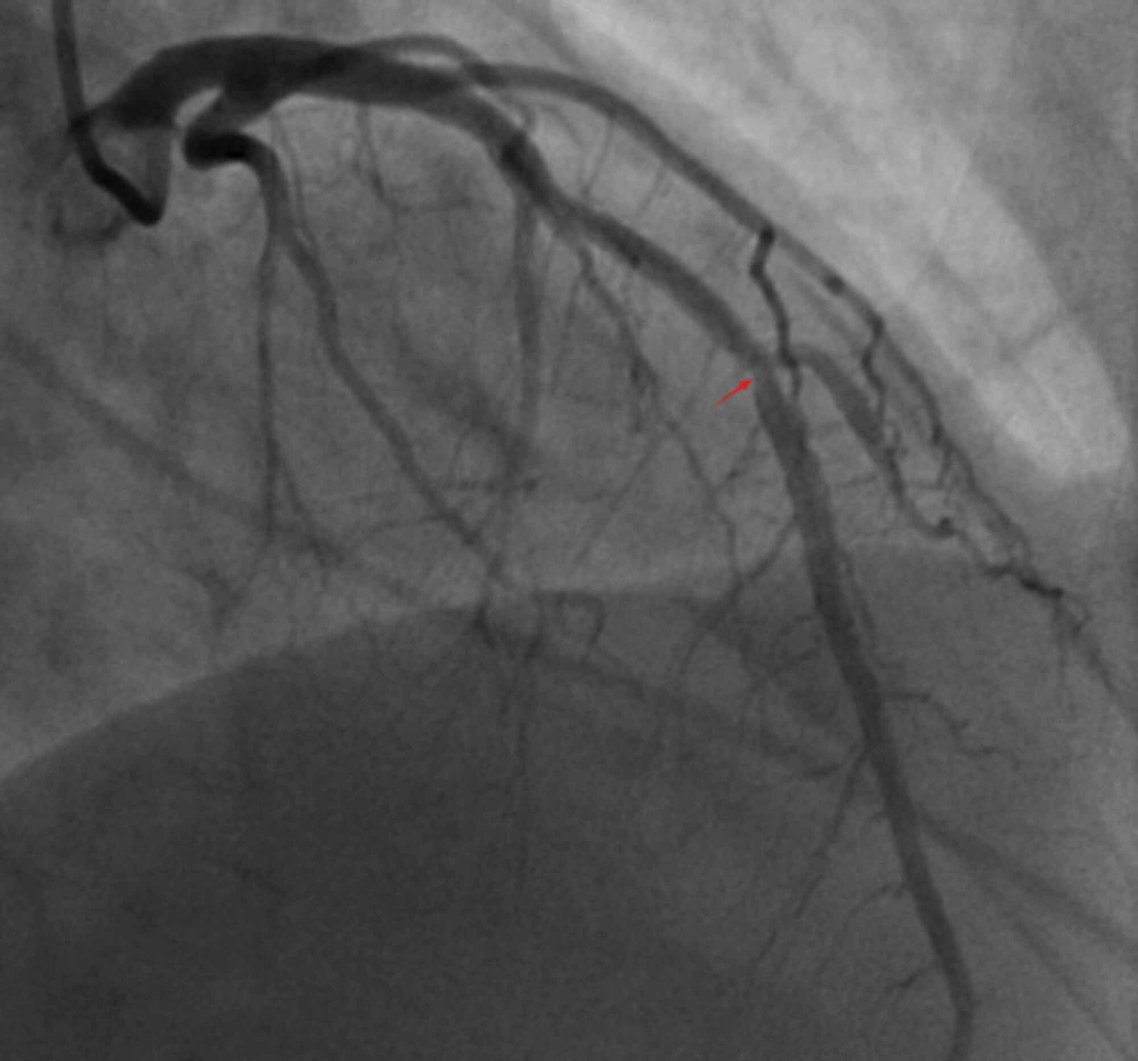
Coronary angiogram showing the mid-LAD lesion in the culprit vessel. Abbreviation: LAD, left anterior descending coronary artery.

Her physical examination was notable for sinus tachycardia with normal S1 and S2 without any additional murmur. Her lungs were clear to auscultation bilaterally. Laboratory workup showed an initial troponin value of 0.062 ng/mL, which later peaked at 20.455 ng/mL. Complete blood count and a comprehensive metabolic profile were completely normal. Her chest X-ray findings were normal without evidence of mediastinal widening. Coronary angiography showed a focal 60% hazy stenotic lesion in the mid-LAD at the site of bifurcation of the diagonal branch. The distal LAD had focal complete occlusion after which it wrapped around the cardiac apex and supplied the inferior wall (Figure 2).

**Figure 3:**
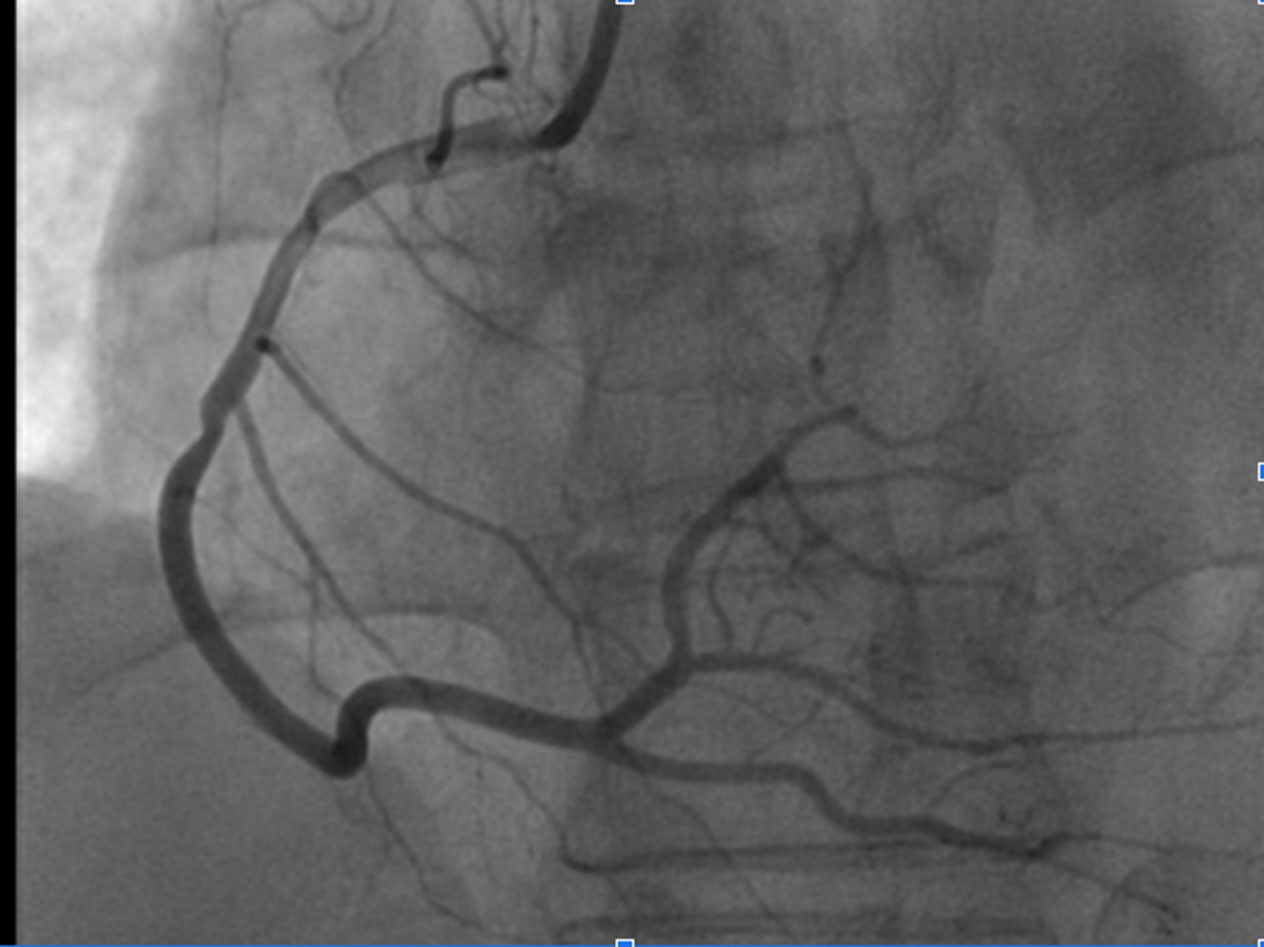
Coronary angiogram showing a dominant RCA with no occlusion in the right system. Abbreviation: RCA, right coronary artery.

The remaining coronary vessels including the RCA appeared angiographically normal (Figure 3).

## Discussion

ECG is an important adjunct in diagnosing patients who present with acute MI. The ECG changes in different leads help to gauge the thrombosed vessels in the setting of acute MI. Simultaneous anterior and inferior MI due to distal LAD occlusion has been described; however, isolated inferior STEMI presentation based on LAD occlusion alone is rarely reported [4]. A “wrapped LAD” is considered an anomalous LAD that wraps around the apex of the heart and perfuses at least one-fourth of the inferior wall of the left ventricle (LV).

In a subgroup of patients who have wrapped LAD, and the location of the occlusion is proximal to the diagonal branch (D1), the ST segment is elevated in the anterior leads and remains iso-electric in the inferior leads. However, in a patient who has wrapped LAD, and the location of the occlusion is distal to the diagonal branch (D1), the ST segment is elevated in the anterior and inferior leads simultaneously. A wrapped LAD usually causes inferior STEMI changes on ECG when the left coronary circulation is dominant (i.e., the LAD gives rise to the posterior descending artery) [5]. There is also literature suggesting that distal LAD occlusion can lead to inferior wall STEMI changes in patients with wrapped LAD [6-8].

Our case is unique and demonstrates that inferior STEMI changes can still be present despite a dominant right system, as was the case in our patient. Several studies have highlighted the importance of identifying the site of the termination of LAD [6]. Inferior wall MI is usually associated with hypotension and bradycardia rather than a hypertensive response, such as the one displayed by our patient on presentation. Another important differential diagnosis to consider is aortic dissection in patients presenting with chest pain and hypertensive urgency [9]. Overall prognosis and mortality are primarily dependent on the myocardial area supplied by the LAD, which is directly proportional to the length of the LAD [10].

A systematic literature search was conducted on PubMed, Medline, and Google Scholar using the Medical Subject Heading (MeSH) terms “wrapped LAD”, “inferior STEMI”, “Anterior STEMI”. The MesH terms were combined using AND or OR. Inclusion criteria were studies written in English or foreign languages translated into English, and articles focused on the management of wrapped LAD describing clinical course and outcomes. Exclusion criteria were studies with no measurable outcomes or clinical follow-up. The reported outcomes of cases similar to ours are displayed in Table 1 [3,4,11-13].

**Table 1:** Outcomes of cases similar to our case Abbreviations: avF, augmented vector foot; STEMI, ST-elevation myocardial infarction.

Author	Year of presentation	Gender	Age (years)	Initial presentation	Leads involved
Kim et al. [3]	February 2019	Female	54	Ventricular fibrillation	II, III, avF, V2-V5
Honda et al. [4]	December 2013	Male	53	Chest pain	ST depressions in V2-V5, STEMI in II, III, avF
Hsu et al. [11]	January 2012	Male	56	Chest pain	STEMI in II, III, avF, V2-V6
Akpinar et al. [12]	July 2008	Male	50	Chest pain	II, III, avF, V2, V3
Roy et al. [13]	June 2014	Male	40	Chest pain	II, III, avF, V5, V6

## Conclusions

Inferior ST-segment elevation in association with anterior lead changes can be a clue to wrapped LAD occlusion in a few rare instances. It is essential for clinicians to look for the underlying etiology and plan to open the occluded vessel promptly.
